# Arsenic content in two-year-old *Acer platanoides* L. and *Tilia cordata* Miller seedlings growing under dimethylarsinic acid exposure–model experiment

**DOI:** 10.1007/s11356-018-04121-x

**Published:** 2019-01-11

**Authors:** Sylwia Budzyńska, Piotr Goliński, Przemysław Niedzielski, Monika Gąsecka, Mirosław Mleczek

**Affiliations:** 1 Department of Chemistry, Poznań University of Life Sciences, Wojska Polskiego 75, 60-625 Poznań, Poland; 2 Faculty of Chemistry, Adam Mickiewicz University, Umultowska 89, 61-614 Poznań, Poland

**Keywords:** Arsenite, Arsenate, Cacodylic acid, Phytoextraction, Trees

## Abstract

The presence of cacodylic acid (dimethylarsinic acid, DMA) can be an important factor in limiting the abilities of young tree seedlings to adapt to unfavorable environmental conditions. For this reason, the aim of the study was to estimate the influence of different DMA additions (from 0.01 to 0.6 mM) to modified Knop solution to arsenic (As) and selected forms of this metalloid (As(III), As(V), DMA) phytoextraction by two-year-old *Acer platanoides* L. and *Tilia cordata* Miller seedlings. Additionally, the biomass and other elements important in As transport in plants were analyzed. Seedlings of both tree species were able to grow in all experimental systems except the one with the highest DMA concentration (0.6 mM). Exposure of tree seedlings was related to a general decrease in plant biomass. Phytoextraction of As in roots, stems, and leaves increased with a rise of DMA concentration in solution to the highest content of As in *A. platanoides* and *T. cordata* roots growing under 0.3 mM (135 ± 13 and 116 ± 14 mg kg^−1^ dry weight). Arsenic was accumulated mainly in roots, thereby confirming bioconcentration factor values BCF > 1 for all tree seedlings treated with DMA. Exposure of plants to low DMA concentrations (0.01 and 0.03 mM) was related to the transport of this element to aboveground parts, while increased DMA concentration in other experimental systems led to the limitation of As transport to stems, as confirmed by translocation factor values TF < 1. Changes in many other elements such as boron, silicon, phosphorus, or sulfur concentration indicated the possible influence of DMA on the transport of As from roots to leaves. The obtained results show that DMA can be an important factor in modulating As phytoextraction in the studied tree species.

**Electronic supplementary material:**

The online version of this article (10.1007/s11356-018-04121-x) contains supplementary material, which is available to authorized users.

## Introduction

The chemistry of arsenic (As) in the environment is complex due to the many different chemical forms of this metalloid that occur (Geiszinger et al. 2002; Upadhyay et al. 2018). Current studies mostly emphasize inorganic As forms which are predominant in aerobic and anaerobic soils, arsenite (As(III)), and arsenate (As(V)), respectively (Li et al. 2016; Abbas et al. 2018), where As(III) is considered as more soluble, mobile, and cytotoxic than As(V), (Chandrakar et al. 2016). On the other hand, the most common organic forms of As in soils are monomethylarsonate (MMA) and dimethylarsinate (DMA), (Fitz and Wenzel 2002; Feldmann et al. 2018). Speciation of As is dynamic because of the inter-conversion of As(III) and As(V) through redox cycling and methylation to organic forms (Abbas and Meharg 2008). Methylation is the process of replacing one or more hydroxyl ligands (-OH) by a methyl group (-CH_3_) in inorganic As structures (Paul et al. 2015) and it is a well-characterized detoxification mechanism in a wide range of organisms from bacteria to mammals and probably in plants also (Abbas and Meharg 2008; Lomax 2013).

According to, e.g., Carbonell-Barrachina et al. (1999), Bergqvist and Greger (2014), or Zhang et al. (2017), inorganic forms of As are treated as more harmful than organic forms to living organisms. It is worth underlining, however, that some researchers have found organic As to be more toxic compared to inorganic forms (Mandal and Suzuki 2002; Yamanaka et al. 2004; Yoon et al. 2015). There is increasing evidence to suggest that methylated metabolites of As cause oxidative stress, cytotoxicity, and may be carcinogenic for exposed organisms (Nigra et al. 2017). The possible higher toxicity of DMA in relation to As(III) or As(V) has been described, e.g., in one of our most recent studies (Budzyńska et al. 2017b, 2018), where two-year-old seedlings of *Ulmus laevis* and *Acer platanoides* were exposed to As(III), As(V), and DMA forms in a pot experiment. *U. laevis* and *A. platanoides* seedlings were not able to survive under DMA, while in the case of inorganic forms, the growth of trees was not disturbed. For this reason, to expand our knowledge of the role of DMA in the soil, the influence of this As form on plant survivability as well as on the phytoextraction of other As forms or other elements, it is necessary to examine the unique toxic properties of DMA.

Information about inorganic compounds has dominated in investigations of As in soils, whereas organic As compounds, which are sometimes found as minor components of soil, can also reach high concentrations (Singh et al. 2015). Methylated As forms have been detected, e.g., in acidic fen soil (Huang and Matzner 2006), in agricultural soil from cotton-producing areas (Bednar et al. 2002), in the orchard, upland, and paddy soils (Takamatsu et al. 1982) as well as in peat (Zaccone et al. 2018). DMA was the second most common organic As species (after arsenobetaine, AsB) in the forest floor, up to 0.006 mg DMA kg^−1^, which was 12% of the total extractable As detected by Huang and Matzner (2007). An interesting fact is that the half-life of DMA is about 20 days and 31 days in untreated and As-amended soils, respectively (Abbas and Meharg 2008).

The mechanisms of As(III) and As(V) uptake and toxicity in plants are generally understood, but those for DMA are less well elucidated (Meharg and Hartley-Whitaker 2002). Abedin et al. (2002) first showed that uptake of DMA has a slow rate, was concentration dependent, and could be described by linear functions and the Michaelis-Menten kinetics model. Additionally, Abbas and Meharg (2008) found a much lower rate of DMA influx in plants when compared to As(III) and As(V), and they confirmed the possibility to describe some parameters using the Michaelis-Menten model. A similar concentration-dependent influx of DMA and well-described uptake by the model mentioned above was shown for rice *Oryza sativa* L. by Rahman et al. (2001).

There is evidence that DMA is a highly mobile form within the plant, more effectively translocated from root to the shoots than other As forms (Faroog et al. 2016). Raab et al. (2007) showed tenfold higher shoot/root translocation factors (TFs) than those of As(V) in 46 investigated plant species from 13 different families. High rates of DMA readily translocated from roots to shoots were also described, e.g., in grass *Spartina alterniflora* by Carbonell et al. (1998a, 1998b), in tomato *Lycopersicum esculentum* by Burló et al. (1999), in radish *Raphanus sativus* by Tlustoš et al. (2002), in pepper *Capsicum annum* L. by Szákova et al. (2007), in turnip *Brassica napus* L. by Carbonell-Barrachina et al. (1999) and Yao et al. (2009), in mustard *Sinapis alba* by Jedynak et al. (2010), and in castor bean *Ricinus communis* by Ye et al. (2010) as well as in rice *O. sativa* L. by Marin et al. (1992) and Carey et al. (2011). Higher translocation of DMA can be explained by the lack of a complex with thiols such as phytochelatin (PC) formation (Raab et al. 2007).

The aim of the study was to determine the phytoextraction efficiency of As and its selected forms (As(III), As(V), and DMA) in two-year-old seedlings of *Acer platanoides* L. and *Tilia cordata* Miller growing in Knop solution enriched with different dimethylarsinic acid (DMA) concentrations. Plants were characterized by their biomass and concentration of particular As forms and calcium (Ca), potassium (K), magnesium (Mg), and sodium (Na) in roots, stems, and leaves of seedlings. Additionally, analyses of boron (B) and silicon (Si) content, elements important in As(III) transport and also phosphorus (P) and sulfur (S), and elements crucial in As(V) transport were performed. This study is a response to the question to our previous work of the real role of DMA to survivability, adaptation, biomass crop, As, and other elements phytoextraction by organs of selected tree species (Budzyńska et al. 2017b, 2018). Presence of DMA in the environment can be an important factor limited to survivability of studied young tree species and finally their use in phytoremediation practice.

## Materials and methods

### Characteristics of experimental materials

Experimental plant materials were obtained from the forest nursery of the Pniewy Forest Division (52° 29′ 4″ N; 16° 15′ 28″ E) on 20 March 2017. Two-year-old leafless seedlings of *A. platanoides* L. (Norway maple) and *T. cordata* Miller (Linden) used in the experiment were characterized by mean biomass of 99.6 ± 3.7 (range from 88.8 to 106.9 g) and 82.5 ± 2.2 g (range from 77.5 to 86.9 g), respectively. All seedlings were planted in the experiment in such a way that the mean biomass of plants growing in a particular experimental system was almost the same.

### Experiment design

Seedlings of *A. platanoides* L. and *T. cordata* Miller were brought from the forest nursery in pots (15 × 15 cm, diameter × height) filled with organic garden soil and removed for 10 days to start leaf growth. During this time, each plant was watered with the same amount of deionized water. Afterwards, the plants were washed with distilled water to remove soil particles from the root systems. The plants were then dried with paper towels and weighed to estimate their biomass before the experiment. The seedlings were planted on 27 March 2017 in white cylindrical pots (21 × 21 cm, diameter × height) filled with 1.5 kg of ultrapure quartz sand (content of SiO_2_ > 97%, pH = 7.18, particle size range was 1–3 mm). One plant was placed in one pot. Seven experimental systems (control; DMA_0.01_; DMA_0.03_; DMA_0.06_; DMA_0.1_; DMA_0.3_ and DMA_0.6_) were prepared for each tree species using the following concentration of DMA ((CH_3_)_2_As(O)OH): 0 (control); 0.01; 0.03; 0.06; 0.1; 0.3; and 0.6 mM in modified Knop solution (Barabasz et al. 2010). 1 L of Knop solution per pot and six plants for each experimental system were used. The reason for the use of such concentrations of DMA was based on the results of our previous studies (Budzyńska et al. 2017b, 2018). The mean values of temperature, moisture, and concentration of CO_2_ were 24.8 ± 2 °C, 43 ± 3%, and 474.7 ± 1.5 ppm, respectively during the 3-month long pot experiment. The plants were automatically watered with deionized water during the experiment to provide seedlings with the support of a similar mean level of the solution. Addition of water in this way allowed us to limit evapotranspiration and an extreme increase of DMA presence in the space of roots.

### Sample preparation and biomass analysis

At the end of the experiment, seedlings of both tree species were carefully washed with deionized water, dried with paper towels, and divided into parts (roots, stems, and leaves) to estimate their final biomass and calculate the percentage biomass increase during the experiment. Next, all parts were dried at 105 ± 2 °C for 48 h (leaves) and 120 h (stems and roots) using an electric drier SLW 53 STD (Pol-Eko, Wodzisław Śląski, Poland). Plant parts from particular experimental systems were then ground for 0.5 min in a Cutting Mill SM 200 (Retsch GmbH, Haan, Germany). Accurately weighed 0.300 ± 0.001 g of dry powder samples of plant parts were placed into a glass flask containing 10 mL of phosphoric acid (1 M) and extracted in an ultrasonic bath (30 min at ambient temperature). The solution was then filtered using a paper filter washed by 200 mL of water. The arsenic species and total As concentration were determined immediately after the extraction procedure.

### Arsenic speciation studies

The procedure of As speciation studies has been described in previous work (Niedzielski et al. 2013). The arsenic forms (As(III), As(V), DMA, respectively) were determined by high performance liquid chromatography with hydride generation atomic absorption spectrometry detection (HPLC-HG-AAS). The determination limits were found at the level of 0.1 mg kg^−1^ for all forms determined. Due to a lack of certified reference materials for As speciation in samples extracted by phosphoric acid, the standard addition method was used for accuracy and traceability studies. Recoveries at the level 80–120% were found as satisfactory.

### Determination of As, B, Ca, K, Mg, Na, P, S, and Si in tree parts

The inductively coupled plasma optical emission spectrometer Agilent 5110 ICP-OES (Agilent, USA) was used in As, B, Ca, K, Mg, Na, P, S, and Si determination. A synchronous vertical dual view (SVDV) of the plasma was accomplished with dichroic spectral combiner (DSC) technology which allows the axial and radial view to be analyzed simultaneously. Common instrumental conditions were applied: radio frequency (RF) power 1.2 kW, nebulizer gas flow 0.7 L min^−1^, auxiliary gas flow 1.0 L min^−1^, plasma gas flow 12.0 L min^−1^, charge coupled device (CCD) temperature − 40 °C, viewing height for radial plasma observation 8 mm, accusation time 5 s, 3 replicates. The content of S for selected samples was checked with a FLASH 2000 analyzer (Thermo Scientific) with FPD detector. Traceability was verified using the standard addition methods, and recoveries at the level of 80–120% were found as satisfactory. General characteristics of fundamental analytical data are present in supplementary data (Table S1).

### Statistical analysis and calculations

The obtained results were analyzed using STATISTICA 12.0 software (StatSoft, USA). To compare biomass, elements, or As form contents in parts of both tree species growing in all experimental systems, one-way analysis of variance (ANOVA) followed by the post-hoc Tukey HSD test was applied. Additionally, to compare both total seedling biomass, biomass of plant parts, or elements accumulated in parts of plants growing under the same experimental system, the significances (^*^p < 0.05, ^**^p < 0.01, ^***^p < 0.001) between plants treated with the same DMA addition were determined using a Student’s test. To show the real potential of seedlings of two tree species growing under particular experimental systems for phytoextraction of As, the total content of this metalloid in the total biomass was calculated taking under consideration As content in particular parts and biomass of roots, stems, and leaves.

To determine the ability of the analyzed tree seedlings to uptake As from Knop solution to the plant, the bioconcentration factor (BCF) was calculated as a ratio of As_total_ in stem and leaves to the concentration of this metalloid in solution. Concerning As transport inside the plants, the translocation factor (TF) was calculated as the ratio of the concentration of As_total_ in aboveground parts and the concentration of this element in roots (Ali et al. 2013).

## Results

Both *A. platanoides* and *T. cordata* seedlings were unable to grow in Knop solution enriched with 0.6 mM of DMA (DMA_0.6_ system). These plants were characterized by their leaves withering within just 5 (*T. cordata*) and 8 (*A. platanoides*) days of the beginning of the experiment.

### Seedling biomass and development

**Fig. 1:**
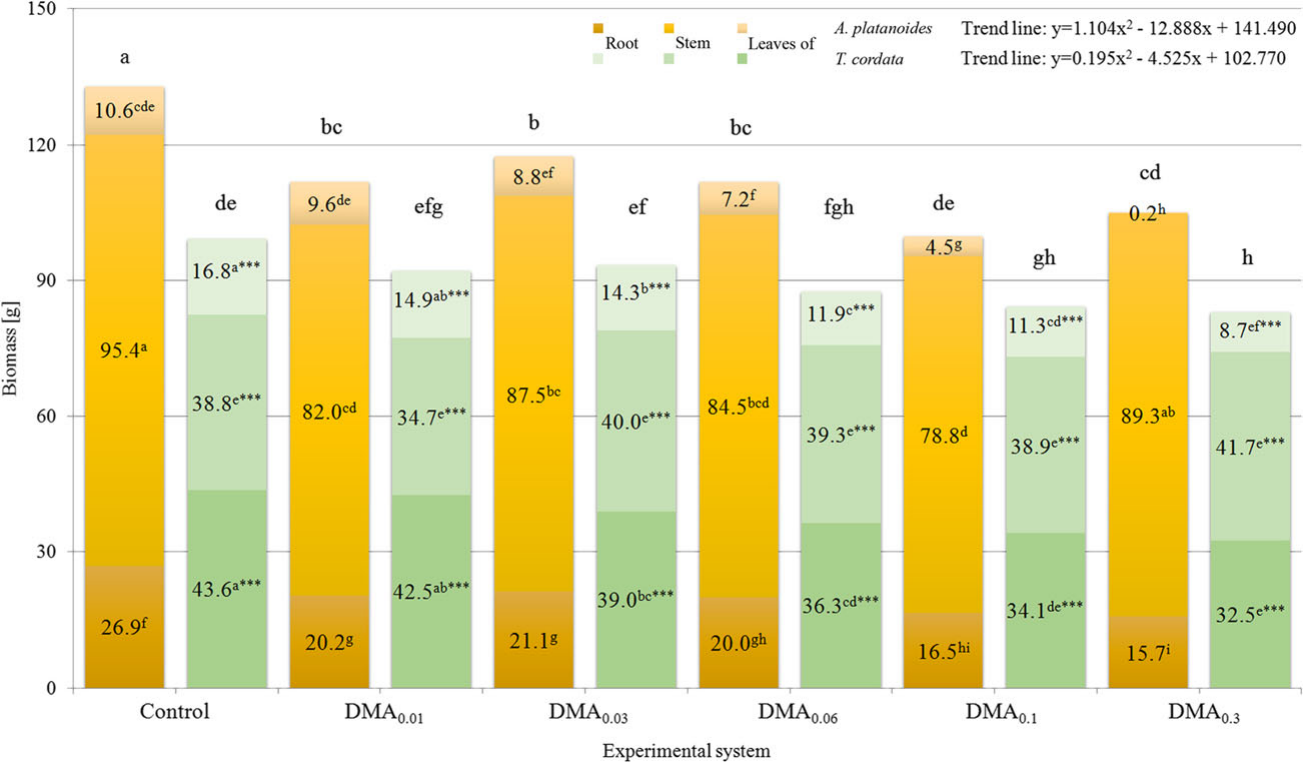
Characteristics of whole biomass and plant parts [g] of *A. platanoides* and *T. cordata* seedlings after experiment

The similar seedling biomass of *A. platanoides* and *T. cordata* planted in particular experimental systems at the beginning had become diverse by the end of the experiment, as shown in Fig. 1. The mean percentage increase of total seedling biomass growing in subsequent systems was as follows: 29.9; 15.4; 16.2; 11.4; 5.2 and 2.9% (*A. platanoides*) and 20.2; 10.9; 10.8; 6.0; 3.0 and 2.3% (*T. cordata*). The highest biomass was characterized by the control seedlings of *A. platanoides* (132.9 g).

In accordance with DMA concentration increase in subsequent Knop solutions (experimental systems), lower increases of plant biomass than those of the control were observed for both tree species. The biomass of *A. platanoides* and *T. cordata* seedlings growing under DMA_0.3_ system (105.1 and 82.9 g, respectively) was characterized by the lowest biomass increase in relation to control plants (132.9 and 99.2 g, respectively), which pointed to the more significant negative influence of DMA on *A. platanoides* than *T. cordata* biomass (79.1 and 83.6% of control seedlings biomass, respectively). These results are reflected in the diversity of biomass of the tree species parts. In the case of *A. platanoides*, the biomass of root, stem, and leaves of plants growing under DMA_0.3_ system was 58.3; 93.6; and 1.7% of control plants, respectively, while for *T. cordata* it was 74.5; 107.5; and 51.8%, respectively of the control. These results have shown that the addition of DMA had a particularly negative influence on *A. platanoides* leaves, while exposure of *T. cordata* to DMA was related to insignificant stem biomass stimulation.

**Fig. 2:**
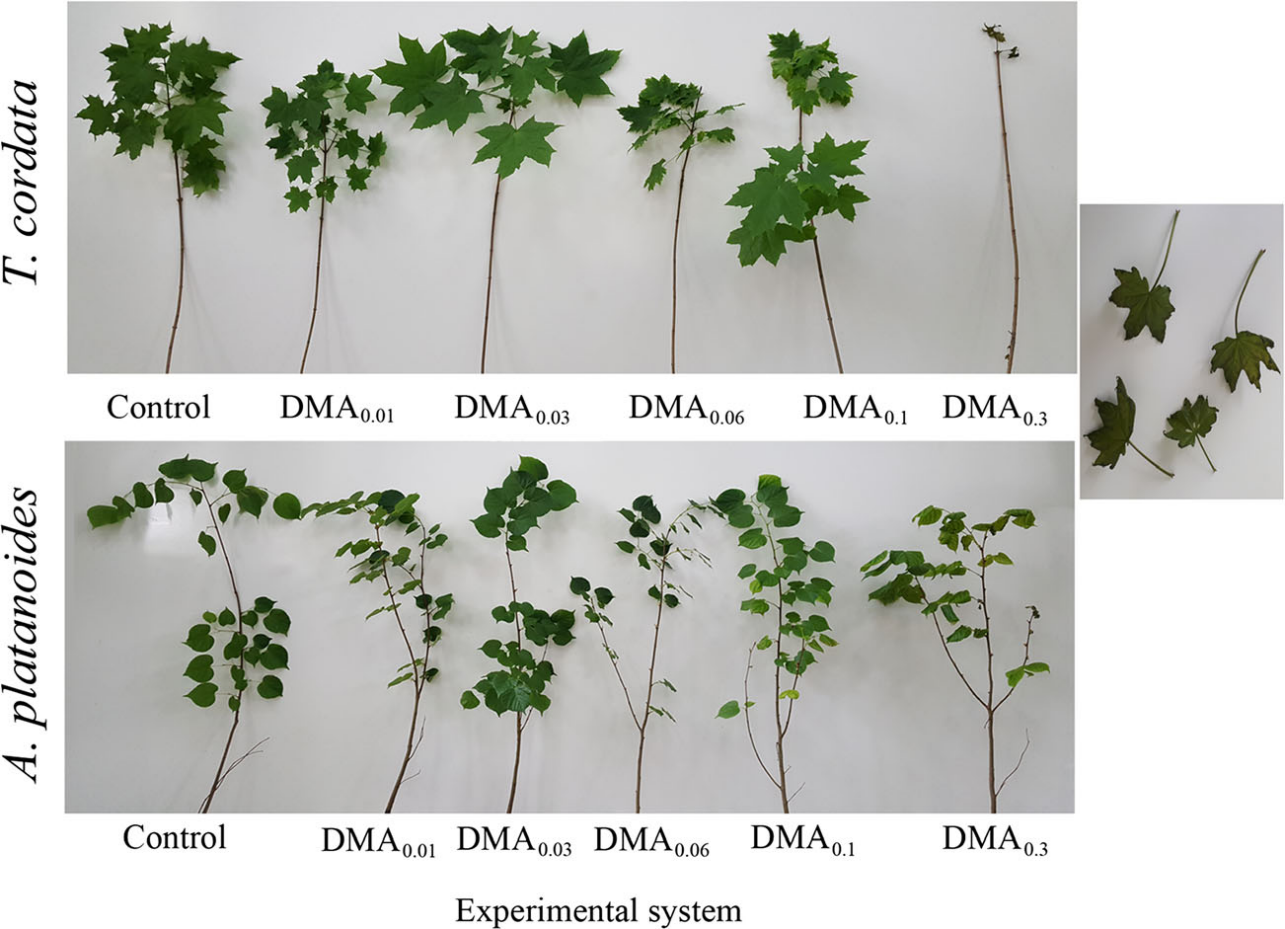
Morphological presentation of seedlings of tree species after experiment

To compare the development of seedlings growing under particular experimental systems, the morphological characteristics of tree seedlings after 90 days of the experiment are shown in Fig. 2. Additionally, characteristics of these plants after 45 days of the experiment (Fig. S1) are presented in Supplementary material.

Negative symptoms of DMA influence were observed after 14 and 19 days from the start of the experiment for *A. platanoides* and *T. cordata*, respectively. It is worth emphasizing that these negative symptoms (brown leaf spots, turning leaves) were characteristic of seedlings of both tree species growing under DMA_0.03_ system only. Moreover, seedlings of both tree species exposed to the DMA_0.03_ system were characterized by almost the same shape and size of leaves, while leaves of plants growing under DMA_0.01_ or DMA_0.06_ systems were smaller in size (Fig. 2).

### Content of As in plant parts

**Fig. 3:**
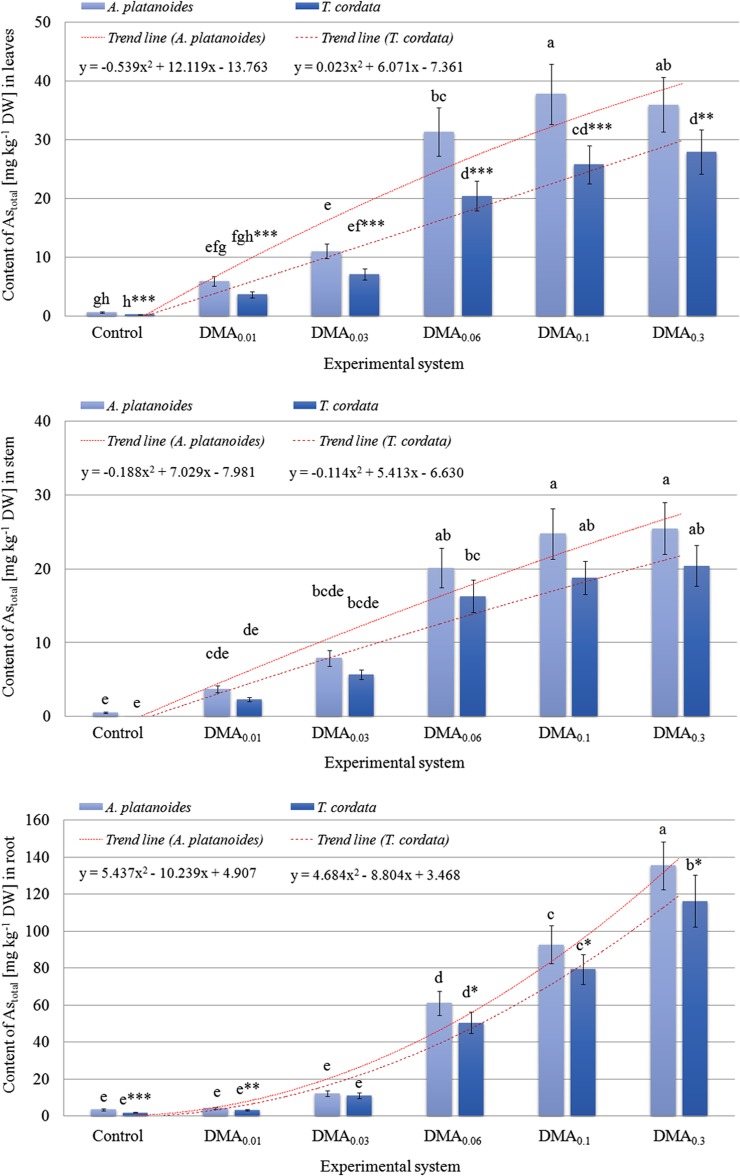
Content of As [mg kg^−1^ DW] in root, stem, and leaves of *A. platanoides* and *T. cordata* seedlings

Exposure of seedlings of the studied tree species to increased DMA concentration in the experimental systems was related to an increase of As content in their roots, stems, and leaves (Fig. 3). Exceptions were leaves of *A. platanoides* and stems of *T. cordata* growing under the DMA_0.3_ system, where an insignificantly lower content of As than for plants under the DMA_0.1_ system was recorded. The highest content of As was observed in *A. platanoides* roots growing under the DMA_0.3_ system (135 ± 13 mg kg^−1^ DW), which was of a significantly higher value than in the roots of *T. cordata* growing under the same experimental system (116 ± 14 mg kg^−1^ DW). A similar tendency for roots of seedlings growing under other experimental systems, except plants exposed to the DMA_0.03_ system, was observed, where the content of As in roots of the two tree species was almost the same. An increase of As content in *A. platanoides* stems was present, while for *T. cordata* similar tendency was observed except plants growing under the DMA_0.3_ system. The opposite situation was noted for leaves, where only *T. cordata* leaves were characterized by a constant increase of As content in the all the subsequent experimental systems. The content of As in *A. platanoides* leaves was significantly higher for seedlings growing in all experimental systems than in *T. cordata* leaves.

Content of As in roots, stems, and leaves of *A. platanoides* growing under the DMA_0.3_ system was 3870, 4633, and 5139%, respectively, while for *T. cordata* it was 6118, 8500, and 9323%, respectively of the As content in parts of control seedlings, which points to the high efficiency of As phytoextraction in the parts of the studied tree species.

**Fig. 4:**
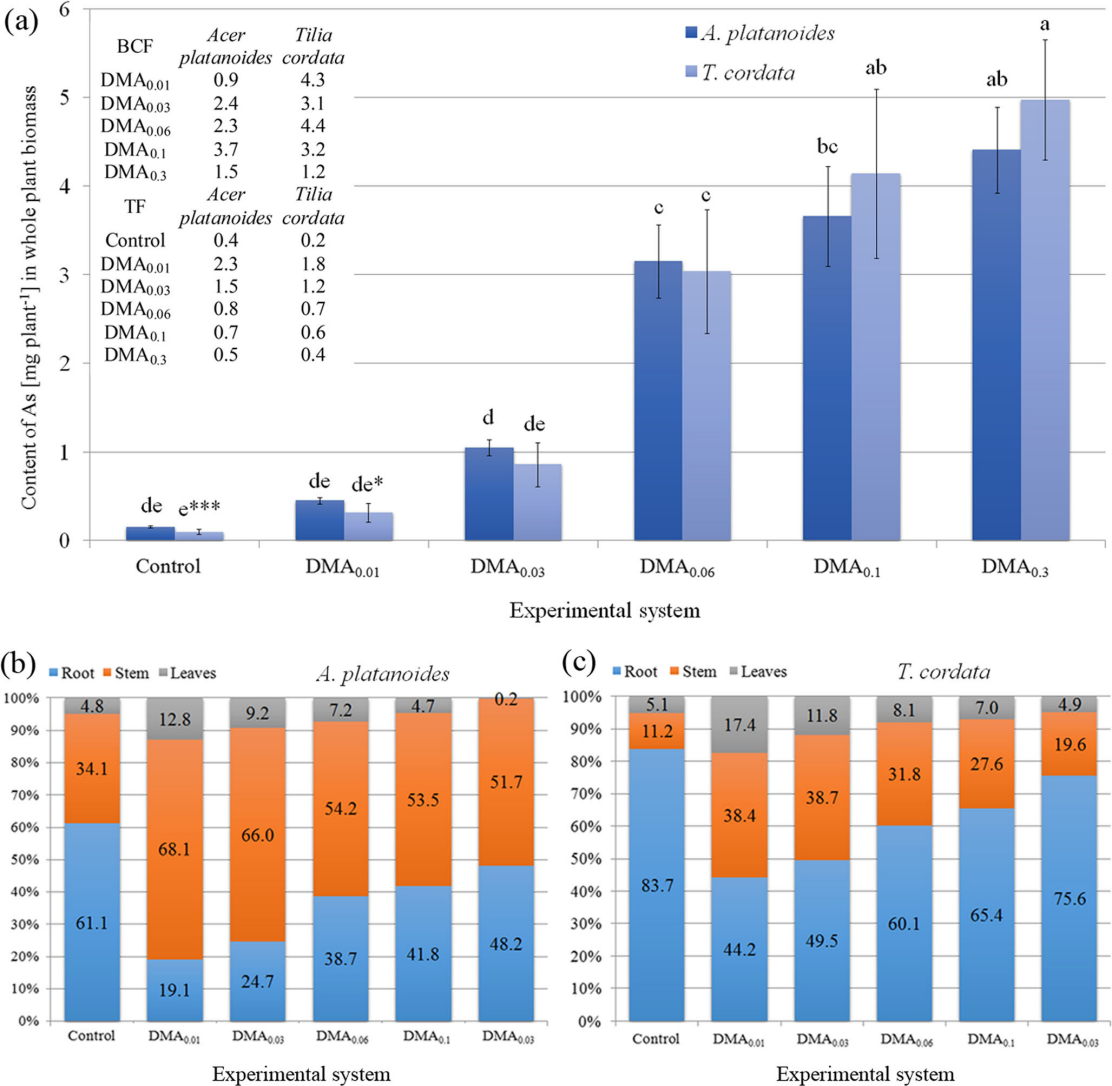
Content of (a) As [mg per plant] with bioaccumulation factor (BCF) and translocation factor (TF) values and percentage distribution of As_total_ in plant parts of (b) *A. platanoides* and (c) *T. cordata*

The content of As in the whole biomass of tree seedlings rose with increased concentration of this metalloid in Knop solution (Fig. 4a). The highest content of As was recorded in *A. platanoides* and *T. cordata* seedlings growing under the DMA_0.3_ system (4.97 ± 0.68 and 4.41 ± 0.49 mg per plant, respectively). An especially significant increase of As content in seedlings of both tree species growing under the DMA_0.03_ and DMA_0.06_ system was observed. The high efficiency of As accumulation confirmed BCF values > 1 for both tree species and experimental systems, except *A. platanoides* seedlings growing under DMA_0.01_. Effective transport of As from the root system to aerial parts of seedlings described by TF > 1 values was only recorded for *A. platanoides* and *T. cordata* growing under DMA_0.01_ and DMA_0.03_.

To show differences in As content in particular parts of plants, the percentage share of As content in parts in relation to As content in the whole plant was calculated separately for *A. platanoides* (Fig. 4b) and *T. cordata* (Fig. 4c).

In control seedlings of both tree species, roots contained the greatest amount of As (61.1 and 83.7% of As_total_, respectively). The addition of the lowest doses of DMA into Knop medium caused As to be transported mainly to stems with only limited As transport to roots, especially for seedlings growing under the DMA_0.01_ and DMA_0.03_ systems. With progressively higher concentrations of DMA, the amount of As deposited in roots became higher. This tendency was similar in *A. platanoides* and *T. cordata* seedlings but with different proportions of As content in particular parts (Fig. 4b, c).

### Characteristics of As forms in tree seedling parts

**Fig. 5:**
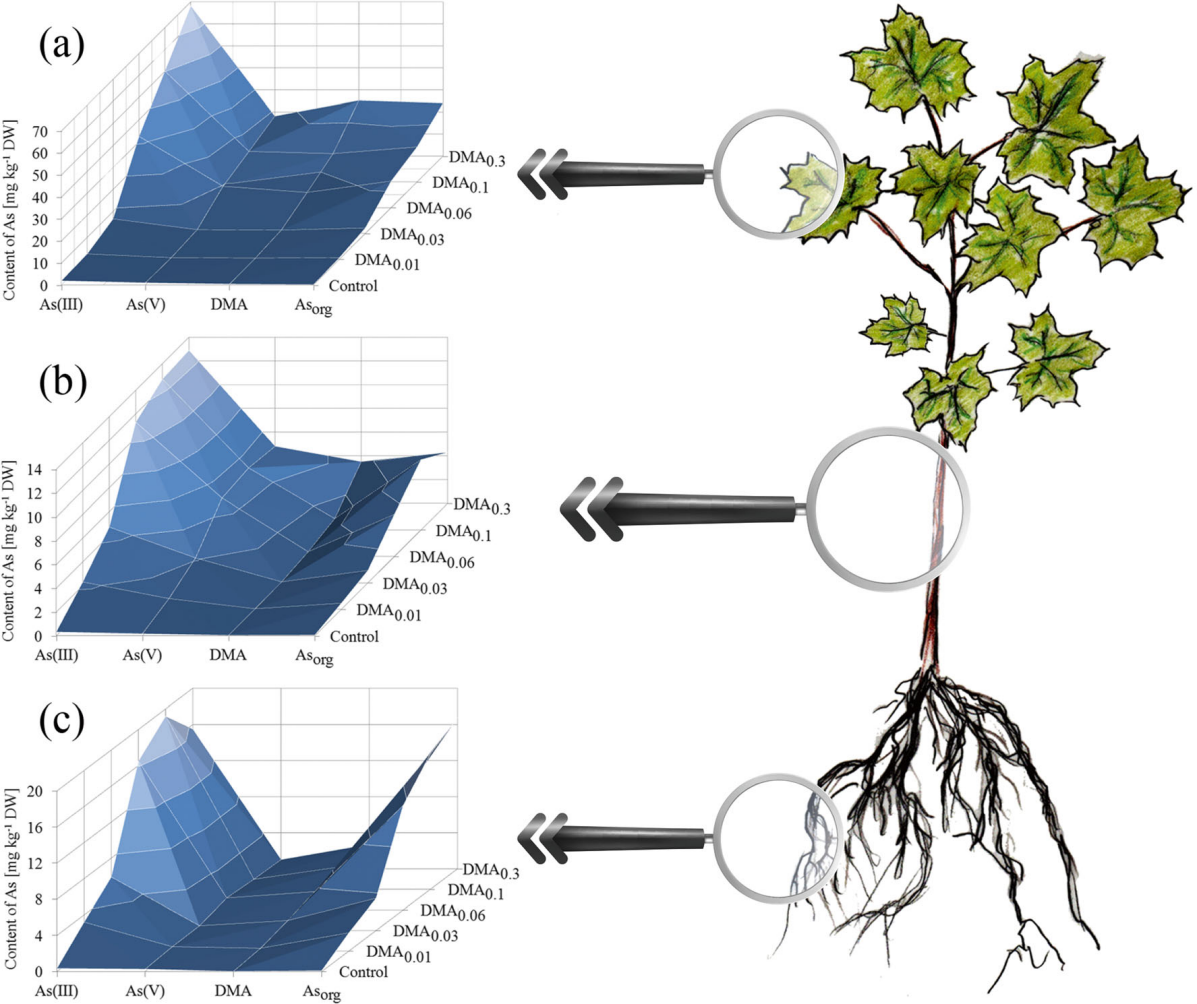
Content of As forms [mg kg^−1^ DW] in (a) leaves, (b) stem, and (c) roots of *A. platanoides* seedlings

Differences in As phytoextraction and transport to aboveground parts of the two tree species are reflected in differences in the content of As forms. Depending on the organ, DMA concentration in solution and tree species, the content of As(III), As(V), and DMA was diverse. In the case of roots of *A. platanoides*, a dominant As form was As(III) with the other forms taking a similar share (Fig. 5). In stems, especially in plants exposed to higher concentrations of DMA (DMA_0.06_, DMA_0.1_, and DMA_0.3_ systems), As(III) was a dominant form with a large share of As_org_ and As(V). In leaves of seedlings growing under these systems, the dominant forms were As(III) and As_org_ (Detailed results are presented in Table S2 of Supplementary material). It is worth emphasizing that the As form added to the medium was DMA, present in roots of *A. platanoides*, while the content of this As form in stem and leaves was marginal when compared to As_total_.

**Fig. 6:**
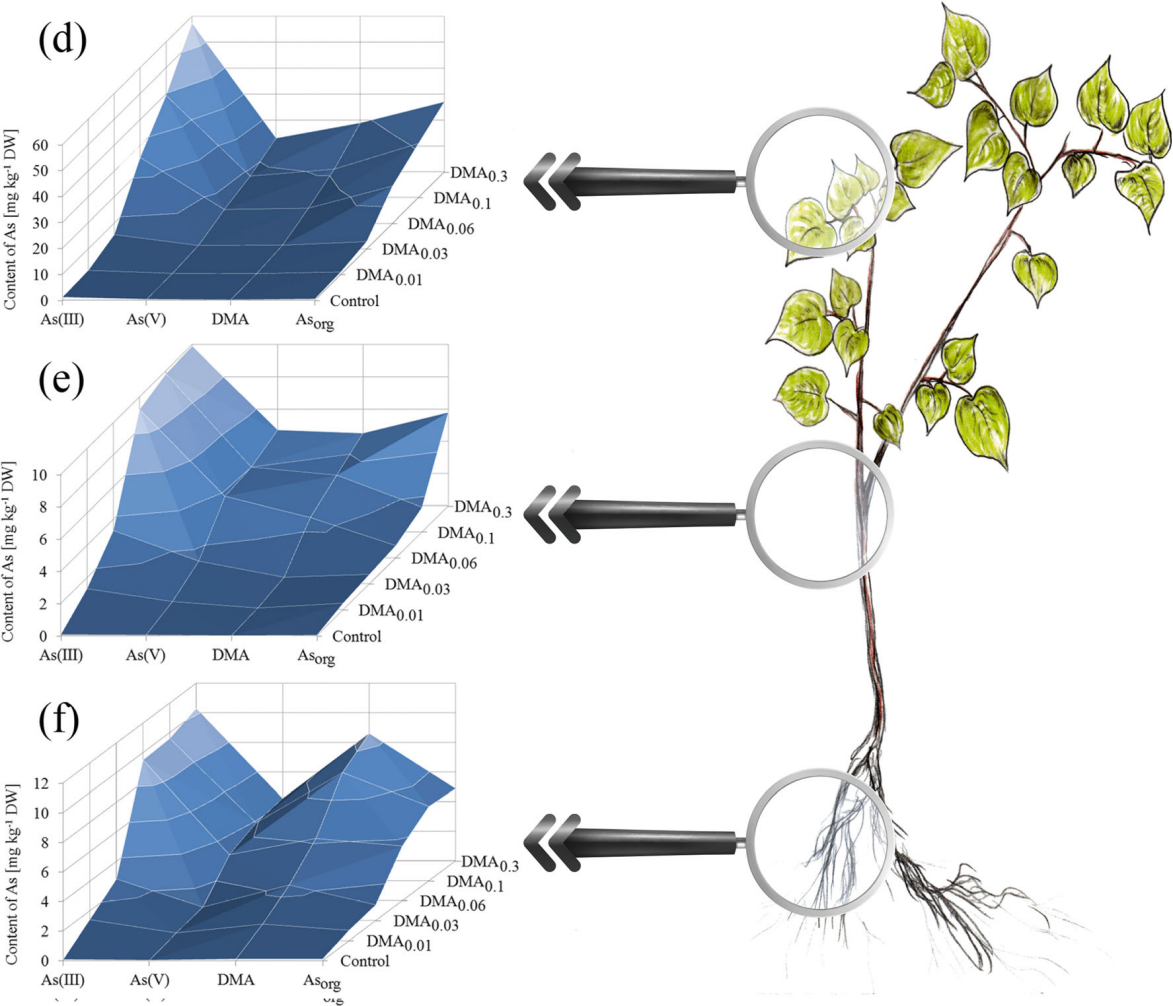
Content of As forms [mg kg^−1^ DW] in (a) leaves, (b) stem, and (c) roots of *T. cordata* seedlings

As(III) was also dominant in the roots of *T. cordata* seedlings but unlike *A. platanoides* seedlings there was an insignificantly lower content of As_org_ and lower content of As(V) and DMA (Fig. 6). Stems of *T. cordata* seedlings contained mainly As(III). Interestingly, the leaves of this tree species were characterized by the presence of all the studied As forms, especially As(III) and DMA but also As(V) and As_org_ in similar low amounts (Table S3 in Supplementary material).

### Characteristics of other elements content in tree species parts

Seedlings of both tree species were also characterized by the content of B, Ca, K, Mg, Na, P, Si, and S in their parts. A full description of the results is presented in Tables S4 and S5 in Supplementary material. The content of B in leaves of *A. platanoides* and *T. cordata* was almost identical, while in the stem and roots of both tree species, it decreased with an increase of DMA concentration in Knop solution (Table S4). Almost the same response was observed in the studied tree species for Na and Si, where a corresponding increase and decrease of the content of these elements in the following experimental systems was recorded in all parts. The content of Ca and K in *A. platanoides* leaves; Ca, K, and Mg in *T. cordata* leaves; and Ca and K in *T. cordata* roots decreased with the increase of DMA addition to solution. The content of Ca, K, and Mg in *A. platanoides* roots rose with an increase of DMA concentration in solution, which was a decidedly opposite tendency to that observed for *T. cordata*. An increase of Ca, K, and Mg in *A. platanoides* stems and a decrease of Ca and Mg content in *T. cordata* stems were also observed (Table S4). It is worth to underline that similarities in the response of both tree species to increased concentrations of DMA in Knop solution were found for P and S (Table S5). The decrease in the content of these elements in roots and stems was noted except S in *A. platanoides* roots, where no significant differences were observed for plants growing in particular experimental systems.

## Discussion

Generally, the presence of As in a substrate is related to an unfavorable influence on plant growth and development (Kumar et al. 2018). Our experiment was designed to examine how DMA can influence young seedlings of selected tree species. Budzyńska et al. (2017b) described the differences in phytoextraction of As and the forms this metalloid during exposure of *U. laevis* seedlings to 21 experimental systems with single, double, and triple addition of As(III), As(V), and/or DMA. Seedlings of *U. laevis* were only unable to grow under exposure to DMA addition at a concentration of 0.6 mM, similarly to the results presented in this paper. The dominant inorganic As forms in aerobic and reducing environment are As(V) and As(III) forms, respectively (Yanitch et al. 2017), however, the share of organic forms of As can be up to 30% of the mobile fraction in the unpolluted forest floor (Huang and Matzner 2007). Both of these forms are toxic to living organisms and can easily enter the food chain (Santra et al. 2013). Organic forms of As, characterized by lower toxicity, can also be present in edible parts of vegetables or other plants (Sadee et al. 2016). MMA and DMA are As forms that have a lower share than inorganic forms in total As concentration in soil (Bakhat et al. 2017), but their toxicity, especially of DMA, is still open to discussion (Suriyagoda et al. 2018).

### Growth of trees under DMA exposition

The presence of As in a plant can cause significant changes in its physiology, morphology, and biochemistry; therefore, in excess, this metalloid negatively affects plant growth and biomass crop (Abbas et al. 2018). The toxic influence of DMA on germination has been described in numerous papers (Duncan et al. 2017), while other papers have suggested that there is no negative influence of this As form (Yoon et al. 2015). Comparison of the As forms most often present is different, e.g., As(V) > As(III) >> DMA for *Zea mays* L. (Mohamed et al. 2008), As(III) > DMA > As(V) for selected cultivated plant species (Yoon et al. 2015), or DMA > As(V) > MMA for *A. thaliana* (Tang et al. 2016). In the case of the latter, toxic influence of As forms was dependent on the time of exposure (DMA > As(V) > MMA in 20-day and MMA > DMA(V) > As(V) in 11-day agar plate experiments), which suggests that toxic effect of DMA is related to plant exposure time. In our 3-month experiment, the toxic influence of DMA could be higher than in the experiment of Tang et al. (2016). Additionally, concentrations of DMA used in the experiments were higher, which can negatively influence both plant biomass and As uptake. Biomass of *A. platanoides* and *T. cordata* seedlings decreased with an increase of DMA concentration in Knop solution. This indicated the negative influence of this As form on plants, but differences between the control and treated seedlings were slight when compared to the effect of this form on other plants, e.g., *Hordeum vulgare* and *Triticum aestivum* (Yoon et al. 2015) or *A. thaliana* (Tang et al. 2016). The lowest biomass of roots and leaves of radish exposed to DMA was described by Tlustoš et al. (2002) with similar As content in both parts. Of course, the radish is not a tree, but it clearly shows the negative influence of DMA on plant growth. A decrease of leaf surface after DMA exposure was described by Marin et al. (1993) in *O. sativa* L. cv. ‘Mercury’, which corresponds to our observation. We have no data on the response of trees to DMA, but our results are similar to many other literature data, where tree species were exposed to As(III) and/or As(V). Each time, a decrease of tree biomass was observed despite varying efficiency of As uptake with an increased gradient in the concentration of this metalloid in the substrate (Yanitch et al. 2017).

### Phytoextraction and translocation of As forms

It is well known that the efficiency of As phytoextraction is mainly dependent on the forms of this metalloid in a substrate, their concentration, bioavailability, and plant species (Carbonell et al. 1998b; Kuehnelt et al. 2000; Tlustoš et al. 2002). One problem of plant response exposed to different DMA concentrations is related to the possible transformation of As forms mainly in roots and stem (Mishra et al. 2017). Exposure of plants to As(V) or MMA is usually related to a greater concentration of As in roots than in aboveground parts, while the opposite situation is observed during DMA presence (Chandra et al. 2018; Ye et al. 2010). In our studies, the highest amount of As was deposited in roots of both tree species, and the dominant form was As(III) in all parts both of *A. platanoides* and *T. cordata*. When comparing the content of As_total_ in parts of these tree species with many other literature data, the described values were relatively high; especially when we take into consideration the slow uptake of DMA in relation to As(III) or As(V) from solution to roots according to the results of Mishra et al. (2017) who found that As phytoextraction was lower when plants were exposed to DMA compared to inorganic As forms.

Exposure of plants to only inorganic As forms is usually related to the presence of inorganic forms in plant tissues (Jedynak et al. 2010); however, Budzyńska et al. (2017a) recorded slight amounts of DMA mainly in roots of *A. platanoides* L., *Betula pendula* Roth., *Quercus robur* L., and *U. laevis* Pall, while Tlustoš et al. (2002) found DMA in roots and leaves of radish. When we compare results described by Raab et al. (2007) for 46 plant species growing under As(V), MMA, and DMA, separately it is quite easy to show that abilities of plants to translocate DMA, generally characterized with higher mobility, are different. Some examples are *Colocasia esculenta*, *Vicia faba* L., *O. sativa* L. var. Bala, or *Thunbergia alata* Bojer ex Sims, where exposure to DMA was each time related with a higher As concentration in roots than in shoots. It worth underlining that Raab et al. (2007) have shown a tendency for all studied plants jointly, as was the case for Ye et al. (2010), who recorded the same pathway of As transport during DMA exposure. Liao et al. (2016) studied interactions between As and phenanthrene in *P. vittata* L. parts found that exposure of plants to As(III) and DMA separately was not caused significantly higher As concentration in pinnae, while exposure to As(V) was related with a significantly higher content of As in this organ. This could be an effect of a significantly higher content of this metalloid in roots.

On the other hand, contents of As_total_ and DMA in tree parts were correlated corresponding to an increase of DMA concentration in Knop solution; the same observation was described by Bakhat et al. (2017). It suggests that the studied tree species effectively accumulated DMA in all parts in proportion to the concentration of this As form in solution. Seedlings of both tree species growing under DMA_0.01_ and DMA_0.03_ systems were characterized by BCF and TF values higher than 1; evidence of the effective uptake of As and translocation of this metalloid to the stem and finally to leaves. Higher concentrations of DMA in Knop solution caused limited As transport to aboveground parts, while uptake of As to roots was still intensive. This points to the influence of DMA on plant biomass (Fig. 2.) and changes in the content of nutritional elements mainly in roots, e.g., potassium in *T. cordata* roots, which in part corresponds to results obtained by Gomes et al. (2012) who studied the savanna tree *Anadenanthera peregrina*.

In the case of rice, Mishra et al. (2017) found the dominant form of As during exposure of *O. sativa* L. to DMA to be just such a form with a marginal share of inorganic As forms in both roots and shoots. We did not observe such a tendency for *A. platanoides* and *T. cordata*, which suggests that As phytoextraction takes place in a different way to many other plant species. Our knowledge about the real nature of DMA and its influence on tree response is still very restricted (Mitra et al. 2017). Abedin et al. (2002) indicated a slow uptake rate of this As form, and a limited description of DMA uptake by Michaelis–Menten kinetics can suggest that this form could affect different plant species in other ways. Slow transport of methylated As forms from the rhizosphere to roots of tree species may be related with their accumulation mainly in roots with the limitation of their transport to aboveground parts such as leaves or needles (Kuehnelt et al. 2000). The possible cause of the lower DMA concentration in leaves than in roots during tree seedling exposure to this As form could also be a high content of organic As forms (As_org_). According to Phillips (1990), methylated As forms can be metabolized to arsenosugars and organo phospholipids, which in the case of the studied tree species was “hidden” as As_org_.

Duncan et al. (2017) have shown that DMA in wheat was transported via Si acid channels and confirmation of this fact was a 40% decrease in Si content. In our experiment, the decrease in Si contents in *A. platanoides* and *T. cordata* were diverse as regards the experimental system. For the highest concentration of DMA (DMA_0.3_ mM system), contents of Si in roots, stems, and leaves were 68, 58, and 59%, respectively of the control in *A. platanoides* seedlings and 66, 53, and 38%, respectively of the control for *T. cordata*, which also suggests the possible use of Si acid channels in these both tree species (Budzyńska et al. 2017a). Abbas et al. (2018) showed organic methylated As forms can be transported from the substrate to plant (xylem) via the Si influx (Lsi1) and Si efflux transporters (Lsi2). Bienert et al. (2008) and Ma et al. (2008) also named OsLsi1 (OsNIP2; 1) as an As transporter in the process of DMA influx.

Interestingly, a decrease in B content was also observed (Table S4). In the case of exposure of *A. platanoides* and *T. cordata* seedlings to the DMA_0.3_ mM system, contents of B in roots, stems, and leaves were 53, 51, 89 and 53, 30, 83%, respectively of those of the control tree species. Such a high decrease may suggest that besides Si transporters, B transporters also participate in DMA uptake from solution. A higher reduction of B and Si content in the *T. cordata* stem was observed than in *A. platanoides*, which could explain the higher content of DMA in aboveground parts of this tree species. Of course, a decrease in B and/or Si content, especially in roots may also be an effect of As(III) presence as the main form of As. On the other hand, such a great decrease in the content of these elements has to be related to the utilization of B and/or Si transporters in DMA uptake. A similar situation was also observed for P and S, elements important in As(V) transport in numerous plants (Zhang et al. 2010; Rasas-Castor et al. 2014). In this case, a decrease in these elements was not as spectacular, which could be the effect of lower contents of just these As forms in tree parts in relation to the other As forms (Figs. 5 and 6). Disturbances in S which plays an important role in binding As(III) to sulfhydryl groups in GSH and PC, together with P (substitution of inorganic phosphate (Pi) by As(V)) can significantly restrict plant development or survivability. Tang et al. (2016) indicated that DMA was more toxic for *A. thaliana* than As(V) or MMA, where a detoxication mechanism is focused on complexation with PC, thiol production, and vacuolar sequestration. In our experiment, there was a decrease of S content in roots and stems with an increase of available DMA, which may suggest that a similar mechanism of thiol production could be characteristic for the studied tree species. Additionally, Tang et al. (2016) found that the described mechanism of detoxification is ineffective for DMA, although the highest used DMA concentration was 0.2 mM, while in our experiment the toxicity was up to 0.6 mM. The similarities in *A. thaliana* and *A. platanoides* or *T. cordata* in plant response and differences in the presence of signs of toxicity are an effect of different plant species and their biomass. In our opinion, the observed changes in P and S content were related more with As(V) presence in plant tissue than negative changes in their metabolism.

## Conclusions

Arsenic, which is becoming an ever-increasing source of environmental pollution, requires the use of an effective method of removal. The share of DMA in numerous post-industrial wastes is slight when compared with the total concentration of As, but the presence of this form may seriously limit the ability of plants in As phytoextraction, growth and development, or even adaptation. In our experiment, DMA caused a general decrease of plant biomass and an increase in As content in parts of *A. platanoides* and *T. cordata* that corresponded with higher concentrations of this As form in solution. Seedlings of both tree species exposed to DMA were characterized by the highest deposition of As in roots and effective uptake of this metalloid, independently of the DMA concentration. On the other hand, the addition of DMA higher than 0.03 mM resulted in significantly more efficient phytoextraction of As with limitation in its transport to aerial parts, as confirmed by TF < 1. The 0.6 mM addition of DMA was lethal for two-year-old tree seedlings, while plants exposed to the same concentration of As(III) or As(V) in solution, were still able to grow, which confirms the findings of our previous studies. It suggests that a higher share of DMA in the total concentration of As in substrate polluted with As can negatively influence the uptake of this element and plant development.

## Electronic supplementary material

ESM 1 (PNG 1428 kb)

High resolution image (TIF 5236 kb)

ESM 2 (DOCX 41 kb)
