# Supplementary figures and images for: Arsenic content in two-year-old Acer platanoides L. and Tilia cordata Miller seedlings growing under dimethylarsinic acid exposure–model experiment
Source: Environ Sci Pollut Res Int. 2019 Jan 11;26(7):6877–89. doi: 10.1007/s11356-018-04121-x (PMC6428799; doi:10.1007/s11356-018-04121-x)

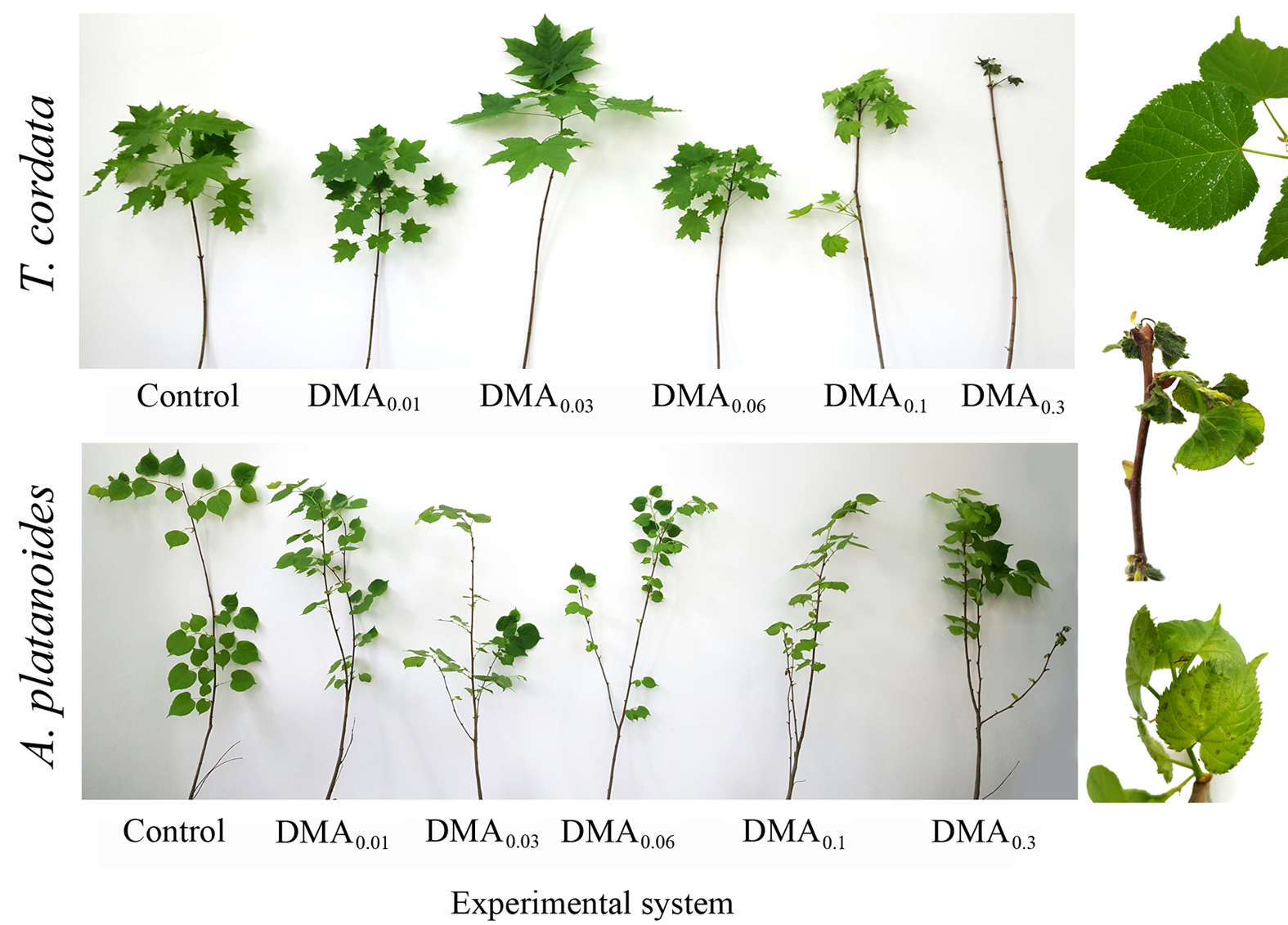

Supplement: Supplementary file 1 — (PNG 1428 kb) [file 11356_2018_4121_Fig7_ESM.png]

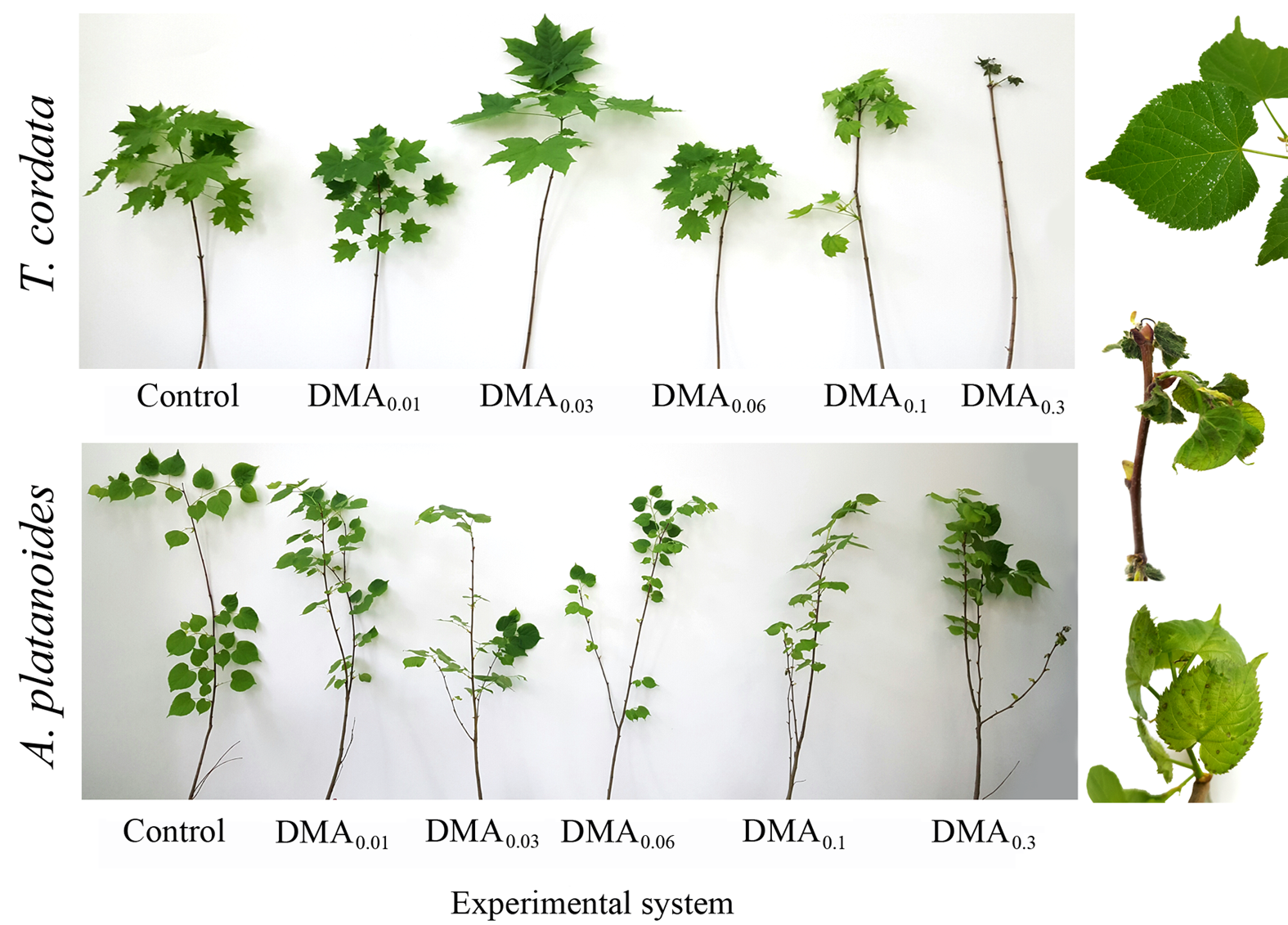

Supplement: Supplementary file 2 — High resolution image (TIF 5236 kb) [file 11356_2018_4121_MOESM1_ESM.tif]
