# Supplementary material for: Arsenic content in two-year-old Acer platanoides L. and Tilia cordata Miller seedlings growing under dimethylarsinic acid exposure–model experiment
Source: Environ Sci Pollut Res Int. 2019 Jan 11;26(7):6877–89. doi: 10.1007/s11356-018-04121-x (PMC6428799; doi:10.1007/s11356-018-04121-x)
Supplement: Supplementary file 3 — (DOCX 41 kb) [file 11356_2018_4121_MOESM2_ESM.docx]

**Supplementary data**

to manuscript: ,,Arsenic content in two-year-old *Acer platanoides* L. and *Tilia cordata* Miller seedlings growing under dimethylarsinic acid exposure – model experiment”

by Budzyńska et al.

Table S1. Characteristics of fundamental analytical data

| Element | Wavelength [nm] | View of plasma | Detection limit  [mg kg^-1^] |
| --- | --- | --- | --- |
| As | 188.980 | axial | 0.01 |
| B | 249.772 | axial | 0.03 |
| Ca | 422.673 | radial | 0.03 |
| K | 766.491 | radial | 0.03 |
| Mg | 285.213 | axial | 0.01 |
| Na | 589.592 | radial | 0.03 |
| P | 253.561 | axial | 0.03 |
| S | 180.669 | axial | 0.04 |
| Si | 288.158 | axial | 0.06 |

Table S2. Content of particular As forms [mg kg^-1^ DW] in *Acer platanoides* L. organs

| Leaves | As_total_ | As(III) | As(V) | DMA | As_org_ |
| --- | --- | --- | --- | --- | --- |
| Control | 0.7^e^ | 0.3^e^ | 0.3^b^ | bDL | 0.1^d^ |
| DMA_0.01_ | 6.0^d^ | 3.2^d^ | 0.5^b^ | 0.3^d^ | 2.0^c^ |
| DMA_0.03_ | 11.1^c^ | 5.9^c^ | 0.5^b^ | 1.1^c^ | 3.5^c^ |
| DMA_0.06_ | 31.4^b^ | 16.5^ab^ | 1.3^a^ | 2.0^b^ | 11.6^b^ |
| D MA_0.1_ | 37.8^a^ | 19.2^a^ | 1.1^a^ | 2.5^a^ | 15.0^a^ |
| DMA_0.3_ | 36.0^ab^ | 15.5^b^ | 1.1^a^ | 2.7^a^ | 16.8^a^ |
| Stem | As_total_ | As(III) | As(V) | DMA | As_org_ |
| Control | 0.6^e^ | 0.3^c^ | 0.2^e^ | bDL | 0.1^e^ |
| DMA_0.01_ | 3.8^d^ | 2.3^b^ | 0.9^d^ | bDL | 0.5^d^ |
| DMA_0.03_ | 7.9^c^ | 4.7^b^ | 2.1^c^ | 0.1^c^ | 1.0^c^ |
| DMA_0.06_ | 20.1^b^ | 11.3^a^ | 4.9^ab^ | 0.4^c^ | 3.5^b^ |
| DMA_0.1_ | 24.8^a^ | 12.7^a^ | 5.2^a^ | 1.0^b^ | 5.9^a^ |
| DMA_0.3_ | 25.5^a^ | 12.9^a^ | 4.2^b^ | 3.5^a^ | 4.3^ab^ |
| Root | As_total_ | As(III) | As(V) | DMA | As_org_ |
| Control | 3.5^d^ | 1.9^e^ | 1.0^cd^ | 0.4^e^ | 0.2^e^ |
| DMA_0.01_ | 4.3^d^ | 2.4^e^ | 0.7^d^ | 0.5^e^ | 0.7^d^ |
| DMA_0.03_ | 12.3^d^ | 6.6^d^ | 1.7^c^ | 1.5^d^ | 2.5^c^ |
| DMA_0.06_ | 61.1^c^ | 32.4^c^ | 9.7^b^ | 6.9^c^ | 12.1^b^ |
| DMA_0.1_ | 92.7^b^ | 50.2^b^ | 13.8^b^ | 12.8^b^ | 15.9^b^ |
| DMA_0.3_ | 135.5^a^ | 68.2^a^ | 18.1^a^ | 25.2^a^ | 24.0^a^ |

n=6; means within columns with different letters (a, b, c…), differ significantly at p≤ 0.05 (Tukey’s HSD test)

bDL - below detection limit

Table S3. Content of particular As forms [mg kg^-1^ DW] in *Tilia cordata* Miller organs

| Leaves | As_total_ | As(III) | As(V) | DMA | As_org_ |
| --- | --- | --- | --- | --- | --- |
| Control | 0.3^e^ | 0.1^c^ | bDL | 0.1^d^ | 0.1^c^ |
| DMA_0.01_ | 3.7^d^ | 1.4^b^ | 0.5^c^ | 1.1^cd^ | 0.7^b^ |
| DMA_0.03_ | 7.2^c^ | 2.8^b^ | 0.9^c^ | 2.4^bc^ | 1.1^b^ |
| DMA_0.06_ | 20.5^b^ | 9.5^a^ | 3.1^b^ | 4.2^b^ | 3.7^a^ |
| DMA_0.1_ | 25.8^ab^ | 9.6^a^ | 3.9^ab^ | 7.3^a^ | 5.0^a^ |
| DMA_0.3_ | 28.0^a^ | 10.3^a^ | 4.2^a^ | 8.6^a^ | 4.9^a^ |
| Stem | As_total_ | As(III) | As(V) | DMA | As_org_ |
| Control | 0.1^e^ | 0.1^e^ | bDL | bDL | bDL |
| DMA_0.01_ | 2.3^d^ | 1.3^d^ | 0.5^c^ | 0.1^c^ | 0.4^c^ |
| DMA_0.03_ | 5.7^c^ | 3.3^c^ | 1.4^c^ | 0.4^c^ | 0.6^c^ |
| DMA_0.06_ | 16.3^b^ | 9.2^b^ | 3.8^b^ | 2.5^b^ | 0.8^bc^ |
| DMA_0.1_ | 18.8^ab^ | 9.8^ab^ | 4.0^ab^ | 3.5^ab^ | 1.5^b^ |
| DMA_0.3_ | 20.4^a^ | 9.9^a^ | 4.7^a^ | 4.5^a^ | 5.8^a^ |
| Root | As_total_ | As(III) | As(V) | DMA | As_org_ |
| Control | 1.9^d^ | 1.1^e^ | 0.3^d^ | 0.1^d^ | 0.4^e^ |
| DMA_0.01_ | 3.3^d^ | 1.6^e^ | 0.4^d^ | 0.3^d^ | 1.0^de^ |
| DMA_0.03_ | 11.0^d^ | 5.5^d^ | 1.3^c^ | 1.4^d^ | 2.8^d^ |
| DMA_0.06_ | 50.5^c^ | 25.3^c^ | 5.2^b^ | 5.7^c^ | 14.3^c^ |
| DMA_0.1_ | 79.3^b^ | 39.4^b^ | 8.2^b^ | 10.9^b^ | 20.8^b^ |
| DMA_0.3_ | 116.3^a^ | 57.3^a^ | 13.0^a^ | 18.9^a^ | 27.1^a^ |

n=6; means within columns with different letters (a, b, c…), differ significantly at p≤ 0.05 (Tukey’s HSD test)

bDL - below detection limit

Table S4. Content [mg kg^-1^ DW] of B, Ca, K, Na, Mg and Si in organs of *A. platanoides* and *T. cordata* growing in particular experimental systems

| Tree species | Tree organ | Experimental system | B | Ca | K | Mg | Na | Si |
| --- | --- | --- | --- | --- | --- | --- | --- | --- |
| *A. platanoides* | Leaves | Control | 35.6±1.4^a^ | 12978±317^a^ | 17851±198^a^ | 4765±124^a^ | 647±34^d^ | 146±10^a^ |
|  |  | DMA_0.01_ | 34.9±2.0^a^ | 11057±249^b^ | 17048±75^b^ | 4897±69^a^ | 843±35^c^ | 134±12^ab^ |
|  |  | DMA_0.03_ | 33.5±0.8^a^ | 9015±203^c^ | 16521±185^c^ | 4902±40^a^ | 1074±50^b^ | 117±8^bc^ |
|  |  | DMA_0.06_ | 33.1±1.4^a^ | 7950±189^d^ | 15033±201^d^ | 4911±58^a^ | 1205±49^a^ | 105±11^cd^ |
|  |  | DMA_0.1_ | 32.4±0.7^a^ | 7429±47^de^ | 14985±168^d^ | 4924±104^a^ | 1173±31^ab^ | 97±6^cd^ |
|  |  | DMA_0.3_ | 31.8±2.1^a^ | 7165±58^de^ | 14629±268^d^ | 4897±96^a^ | 1149±41^ab^ | 86±8^d^ |
|  | Stem | Control | 45.3±5.5^a^ | 12946±186^d^ | 579±47^d^ | 1423±86^d^ | 899±47^b^ | 613±42^a^ |
|  |  | DMA_0.01_ | 43.8±4.3^ab^ | 13597±107^c^ | 994±48^c^ | 1675±119^cd^ | 941±30^ab^ | 537±21^b^ |
|  |  | DMA_0.03_ | 39.5±1.8^abc^ | 14983±112^b^ | 1504±83^b^ | 1849±83^bc^ | 986±41^ab^ | 493±24^bc^ |
|  |  | DMA_0.06_ | 35.9±3.0^bcd^ | 16429±170^a^ | 2081±101^a^ | 2072±101^ab^ | 1032±58^a^ | 429±28^cd^ |
|  |  | DMA_0.1_ | 33.2±2.1^cd^ | 16513±100^a^ | 2105±66^a^ | 2095±81^ab^ | 1054±42^a^ | 398±20^de^ |
|  |  | DMA_0.3_ | 27.6±1.8^d^ | 16486±72^a^ | 2098±89^a^ | 2134±100^a^ | 1013±20^a^ | 356±14^e^ |
|  | Root | Control | 135±4^a^ | 6659±88^d^ | 1058±66^d^ | 279±26^c^ | 128±9^d^ | 13282±304^a^ |
|  |  | DMA_0.01_ | 130±19^a^ | 7947±113^c^ | 1246±46^c^ | 328±19^bc^ | 159±14^cd^ | 11521±259^b^ |
|  |  | DMA_0.03_ | 100±6^ab^ | 10959±366^b^ | 1428±42^b^ | 409±46^ab^ | 186±19^bc^ | 10875±193^c^ |
|  |  | DMA_0.06_ | 85±29^b^ | 17482±351^a^ | 1512±50^ab^ | 498±34^a^ | 234±26^ab^ | 10159±92^d^ |
|  |  | DMA_0.1_ | 73±11^b^ | 17529±186^a^ | 1569±78^ab^ | 476±24^a^ | 242±23^a^ | 9574±170^e^ |
|  |  | DMA_0.3_ | 72±8^b^ | 17493±90^a^ | 1592±59^a^ | 469±42^a^ | 238±13^a^ | 9062±80^e^ |
| Tree species | Tree organ | Experimental system | B | Ca | K | Mg | Na | Si |
| *T. cordata* | Leaves | Control | 213±8^a^ | 5276±66^bc^ | 4679±59^a^ | 4526±193^a^ | 495±20^c^ | 234±22^a^ |
|  |  | DMA_0.01_ | 207±34^a^ | 8991±121^a^ | 4584±324^a^ | 2967±83^c^ | 856±22^b^ | 149±15^b^ |
|  |  | DMA_0.03_ | 165±17^a^ | 6820±450^b^ | 3640±169^b^ | 3765±312^b^ | 967±49^a^ | 132±12^b^ |
|  |  | DMA_0.06_ | 164±43^a^ | 5248±734^bc^ | 3269±317^bc^ | 2965±222^b^ | 1045±48^a^ | 128±17^bc^ |
|  |  | DMA_0.1_ | 180±20^a^ | 3951±611^cd^ | 2864±90^c^ | 3568±127^c^ | 1006±57^a^ | 114±8^bc^ |
|  |  | DMA_0.3_ | 177±33^a^ | 3102±157^d^ | 2216±236^d^ | 3907±97^b^ | 996±21^a^ | 89±6^c^ |
|  | Stem | Control | 134±6^a^ | 3992±37^a^ | 1341±34^a^ | 1318±36^c^ | 587±30^b^ | 795±43^a^ |
|  |  | DMA_0.01_ | 121±10^a^ | 3915±181^ab^ | 1541±92^a^ | 1543±74^b^ | 973±37^a^ | 638±64^b^ |
|  |  | DMA_0.03_ | 97±27^ab^ | 3742±529^ab^ | 1328±365^a^ | 1637±86^ab^ | 876±24^a^ | 592±20^b^ |
|  |  | DMA_0.06_ | 76±14^bc^ | 3111±286^bc^ | 1535±177^a^ | 1769±69^a^ | 956±41^a^ | 471±20^c^ |
|  |  | DMA_0.1_ | 52±10^c^ | 2591±334^c^ | 1353±201^a^ | 1752±56^a^ | 958±44^a^ | 420±15^c^ |
|  |  | DMA_0.3_ | 40±4^c^ | 1776±147^d^ | 1786±148^a^ | 1768±54^a^ | 941±40^a^ | 397±32^c^ |
|  | Root | Control | 177±5^a^ | 8134±41^a^ | 2754±102^b^ | 354±20^b^ | 139±8^b^ | 12083±208^a^ |
|  |  | DMA_0.01_ | 169±3^a^ | 7607±465^ab^ | 3688±221^a^ | 372±21^b^ | 165±9^b^ | 11005±128^b^ |
|  |  | DMA_0.03_ | 159±18^ab^ | 6287±101^bc^ | 3561±276^ab^ | 406±18^b^ | 175±14^b^ | 10037±215^c^ |
|  |  | DMA_0.06_ | 154±40^ab^ | 5592±660^cd^ | 1831±427^c^ | 473±21^a^ | 254±22^a^ | 9508±163^d^ |
|  |  | DMA_0.1_ | 107±8^bc^ | 4084±359^de^ | 1576±370^c^ | 396±26^b^ | 236±18^a^ | 8365±209^e^ |
|  |  | DMA_0.3_ | 93±21^c^ | 3652±583^e^ | 1483±344^c^ | 395±18^b^ | 225±18^a^ | 8006±193^e^ |

Mean values (n = 6); identical superscripts (a, b, c…) denote no significant (p<0.05) difference between mean values in column according to Tukey’s HSD test (ANOVA)

Table S5. Content of P [g kg^-1^ DW] and S [%] in root and stem of *A. platanoides* and *T. cordata* growing in particular experimental systems

| Tree species | Experimental system | Root | | Stem | |
| --- | --- | --- | --- | --- | --- |
|  |  | P | S | P | S |
| *A. platanoides* | Control | 0.81±0.05^a^ | 0.74±0.03^a^ | 0.90±0.04^a^ | 0.69±0.01^a^ |
|  | DMA_0.01_ | 0.75±0.04^ab^ | 0.69±0.02^ab^ | 0.87±0.03^a^ | 0.66±0.03^a^ |
|  | DMA_0.03_ | 0.73±0.02^ab^ | 0.67±0.02^abc^ | 0.84±0.03^a^ | 0.62±0.03^a^ |
|  | DMA_0.06_ | 0.71±0.03^ab^ | 0.66±0.03^bc^ | 0.83±0.05^a^ | 0.54±0.04^b^ |
|  | DMA_0.1_ | 0.70±0.04^b^ | 0.64±0.03^bc^ | 0.83±0.06^a^ | 0.54±0.02^b^ |
|  | DMA_0.3_ | 0.65±0.03^b^ | 0.61±0.04^c^ | 0.80±0.04^a^ | 0.52±0.04^b^ |
| *T. cordata* | Control | 0.87±0.03^a^ | 0.74±0.03^a^ | 0.89±0.03^a^ | 0.66±0.03^a^ |
|  | DMA_0.01_ | 0.81±0.04^ab^ | 0.70±0.04^ab^ | 0.84±0.03^ab^ | 0.63±0.03^ab^ |
|  | DMA_0.03_ | 0.74±0.06^bc^ | 0.65±0.06^bc^ | 0.84±0.04^ab^ | 0.58±0.03^bc^ |
|  | DMA_0.06_ | 0.68±0.05^c^ | 0.64±0.05^bc^ | 0.80±0.04^abc^ | 0.51±0.02^cd^ |
|  | DMA_0.1_ | 0.65±0.03^c^ | 0.62±0.03^bc^ | 0.75±0.03^bc^ | 0.47±0.02^d^ |
|  | DMA_0.3_ | 0.64±0.02^c^ | 0.58±0.02^c^ | 0.70±0.05^c^ | 0.45±0.03^d^ |

Mean values (n = 6); identical superscripts (a, b, c…) denote no significant (p<0.05) difference between mean values in column according to Tukey’s HSD test (ANOVA)

**Figure caption:**

Fig. S1. Characteristics of whole biomass and organs [g] of *A. platanoides* and *T. cordata* seedlings after 45 days of the experiment
